# The association of adiponectin with metabolic syndrome and clinical outcome in patients with non-diabetic chronic kidney disease

**DOI:** 10.1371/journal.pone.0220158

**Published:** 2019-07-19

**Authors:** I-Ching Kuo, Ping-Hsun Wu, Hugo You-Hsien Lin, Sheng-Wen Niu, Jiun-Chi Huang, Chi-Chih Hung, Yi-Wen Chiu, Hung-Chun Chen

**Affiliations:** 1 Graduate Institute of Clinical Medicine, College of Medicine, Kaohsiung Medical University, Kaohsiung, Taiwan; 2 Division of Nephrology, Department of Internal Medicine, Kaohsiung Medical University Hospital, Kaohsiung Medical University, Kaohsiung, Taiwan; 3 Department of Internal Medicine, Kaohsiung Municipal Ta-Tung Hospital, Kaohsiung Medical University, Kaohsiung, Taiwan; 4 Department of Internal Medicine, Kaohsiung Municipal Hsiao-Kang Hospital, Kaohsiung Medical University, Kaohsiung, Taiwan; University of Mississippi Medical Center, UNITED STATES

## Abstract

Adiponectin is the most abundant circulating adipokine, and it has insulin-sensitizing and anti-inflammatory properties. Although it has been speculated that kidney function decline associated with elevated adiponectin is attributable to decreased renal clearance and compensatory responses to adiponectin resistance, it is unclear how elevated adiponectin affects clinical outcomes in chronic kidney disease (CKD) patients and whether the effects are the same as those in the general population. Therefore, the aim of this study is to examine whether the association between serum adiponectin levels and clinical outcomes in non-diabetic CKD patients is independent of adiposity and metabolic syndrome. We enrolled 196 non-diabetic CKD patients with eGFR ranging between 10 and 60 mL/min/1.73 m^2^, these patients were divided into two groups based on the presence of metabolic syndrome. The primary endpoint was all-cause mortality or renal events (renal failure requiring renal replacement therapy [RRT] or 50% reduction in eGFR). During the mean follow-up period of 5 years, 48 (24.5%) incident cases of end-stage renal disease (ESRD) were observed, and 33 (16.8%) deaths occurred. The mean eGFR was 29.8 ± 12.8 mL/min/1.73m^2^. The baseline median adiponectin concentration in the cohort was 29.4(interquartile range, 13.3–108.7) μg/ml. Adiponectin levels were inversely related to body mass index (BMI) (*r* = −0.29; *P* < 0.001) and waist circumference (*r* = −0.35; *P* < 0.001). In the fully adjusted Cox regression model, the hazard ratios (HRs) were 2.08 (95% confidence interval [CI], 1.08–4.02; P = 0.03) for RRT and 1.66 (95% CI, 1.03–2.65; P = 0.04) for composite renal outcome. The risks remained consistent within different subgroups. However, no association was observed with mortality risk. In conclusion, higher adiponectin levels are associated with a higher risk of ESRD independent of conventional risk factors, BMI, and metabolic syndrome components.

## Introduction

Adiponectin is the most abundant adipocyte-synthesized adipokine, circulating in the blood as various multimers, and is expressed at low levels in obese individuals. In contrast to the proinflmmatory properties of most adipokines, adiponectin exhibits insulin-sensitizing, anti-inflammatory, anti-atherogenic, cardioprotective, and anti-oxidative stress effects[1–5]. Adiponectin levels are strongly determined by body composition and are mainly inversely associated with visceral fat, total body fat, body mass index (BMI), and waist circumference[6–10]. As visceral obesity, which often accompanies metabolic syndrome, is a risk factor for the onset and progression of chronic kidney disease (CKD), systemically circulating adipokines may be involved in the pathogenesis of CKD. Studies have indicated that in the general population, lower adiponectin levels are linked to insulin resistance, type 2 diabetes mellitus (DM), and coronary artery disease (CAD) [11–13], which are conditions that may contribute to CKD development.

Dysregulated metabolism of adipokines in the CKD population might be probably due to decreased renal excretion and the effects of chronic inflammation or protein-energy wasting (PEW). Adiponectin levels are markedly elevated with declining kidney function[14], and it possesses significantly negative correlation with eGFR[15–18] despite the presence of high cardiovascular (CV) risks and mortality. In fact, the clinical role of adiponectin is more complex because of varying disease circumstances and the multiplicity of metabolic pathways among CKD patients. Contrary to the beneficial effects of adiponectin in the general population, higher rather than lower adiponectin levels are associated with higher progression of kidney disease[19, 20]. Conflicting results have been obtained regarding the effect of adiponectin on CV outcomes and mortality across studies in the CKD population. High adiponectin levels are related to CV events and all-cause mortality in stage 3–4 CKD or HD patients [6, 21, 22], whereas some studies have found that the association is only dependent on BMI or waist circumference[7, 23]. Besides, as epicardial adipose tissue (EAT) thickness increases in CKD, secretion of adiponectin from EAT also plays the emerging role in cardiac metabolism regulating CV outcomes[24]. Moreover, the largest cohort study showed no significant association between longitudinal changes in adiponectin and mortality in HD patients[25].

The clinical significance of metabolic syndrome in CKD has been clarified; that is, metabolic syndrome is associated with hypoadiponectinemia, insulin resistance, and eGFR decline[10, 26]. In addition, clustering metabolic syndrome components increased the risk of rapid decline of eGFR in a gradual manner[27]. Based on the aforementioned reports, we conducted this cohort study to examine whether the predictive role of adiponectin in ESRD and all-cause mortality in non-diabetic patients with advanced CKD is independent of adiposity, especially BMI, and metabolic syndrome components.

## Materials and methods

### Participants and data collection

This observational study was conducted in the affiliated hospital of Kaohsiung Medical University in Southern Taiwan from January 2010 to June 2017. Patients aged between 20 and 85 years were initially screened from an integrated or traditional CKD care program which included various causes of pre-dialysis CKD. A total of 196 non-diabetic CKD patients with eGFR in the range of 10–60 mL/min/1.73m^2^ were recruited. eGFR was determined using the four-variable Modification of Diet in Renal Disease (MDRD) formula, which is generally applied in the Taiwan National Database to assess CKD prevalence and dialysis initiation[28, 29]. Eligible patients were followed up for more than 3 months to confirm the presence of CKD. Exclusion criteria were as follows: malignancy, liver disease, and active infectious disease. Demographic details, relevant medical history, blood pressure and anthropometric measurements (weight, height, and waist circumference) were recorded by trained nurses in the clinic at the time of enrollment. BMI was calculated as weight in kilograms divided by the square of height in meters (kg/m^2^). All participants provided written informed consent with the approval of the Kaohsiung Medical University Hospital Institutional Review Board (Institutional Review Board number: KMUH-IRB-990198), in accordance with the Declaration of Helsinki.

### Laboratory measurements

Within 1 month of enrollment, blood and urine were sampled from patients after an overnight fast. Blood was drawn into EDTA tubes, centrifuged at 4°C immediately, and then stored at −80°C in aliquots until further analysis. The hemogram and biochemical parameters, including blood sugar, serum creatinine, albumin, HbA1c, phosphate, uric acid, total cholesterol, triglyceride (TG), low-density lipoprotein (LDL-C), high-density lipoprotein (HDL-C), C-reactive protein (CRP), and urine protein-to-creatinine ratio (UPCR) were measured using an automated analyzer following standardized procedures at the Department of Laboratory Medicine at Kaohsiung Medical University Hospital. According to the modified National Cholesterol Education Program Adult Treatment Panel III (NCEP-ATP III) criteria, metabolic syndrome was defined as the presence of three or more of the following components: (1) central obesity (waist circumference ≥ 90 cm in men or ≥ 80 cm in women, according to the ethnic criteria for Asians); (2)TG ≥ 150 mg/dL or receiving treatment with fibrates; (3) HDL cholesterol < 40 mg/dL in men and < 50 mg/dL in women or receiving treatment with statins; (4) fasting glucose ≥ 100 mg/dL; and (5) BP ≥ 130/85 mmHg or receiving treatment with antihypertensive drugs[30]. Plasma adiponectin concentrations were determined using a MILLIPLEX MAP Human Adipokine Magnetic Bead Panel 1 kit (Millipore, Billerica, MA). The coefficient of variability for intra- and inter-assay precision were 7.3% and8.1%, respectively.

### Definition of clinical outcomes

The primary endpoint was all-cause mortality and composite renal outcomes (ESRD or 50% reduction in eGFR from baseline). All-cause mortality was determined through death certificates and the National Death Index. ESRD was defined as a history of renal replacement therapy (RRT) (commencement of hemodialysis, peritoneal dialysis, or renal transplantation), which was ascertained using catastrophic illness cards issued by the Bureau of National Health Insurance. For patients who reached the renal endpoint, their eGFR data were collected until dialysis initiation. Other patients periodically received follow-up until June 30, 2017, and during follow-up, they underwent serial blood examination and evaluation of CKD complications.

### Statistical analysis

Descriptive statistics are summarized as frequency and percentage for categorical data, as means ± standard deviation (SD) for continuous variables with normal distributions, and as median with interquartile range (25^th^–75^th^ percentile) for non-normally distributed data. Normal distribution was tested using the Kolmogorov–Smirnov test. Between-group comparisons were achieved by chi-square test for categorical variables, the t-test for normally distributed continuous variables, and the Mann-Whitney U test for variables with non-normal distributions. To determine the variables which were independently predictive of adiponectin levels, multivariable linear regression analysis was employed including five metabolic indicators (waist circumference, TG, HDL-C, HTN, and fasting glucose). Logarithmic transformation of variables with a skewed distribution (TG, UPCR, and CRP) was applied in analysis.

The multivariate Cox proportional hazards model was used to assess the association of baseline variables with clinical outcome risks through estimating the hazard ratio (HR) and corresponding 95% confidence interval (CI) of each predictor. Sequential models were established with three incremental levels of covariates: (1) unadjusted analysis; (2) model 1 was adjusted for age, sex, gout history, and BMI; (3) model 2 was adjusted for model 1 covariates as well as metabolic syndrome components; and (4) model 3 was adjusted for model 2 variables, as well as other laboratory potential confounders such as albumin, hemoglobin (Hb), CRP, eGFR, and UPCR, which were selected based on previous publications and clinical relevance. We subsequently conducted subgroup analyses stratified by age (≥65 vs <65 years), sex, eGFR (≥25 vs <25 mL/min/1.73m^2^), UPCR (≥ 0.5 vs <0.5 g/gCr), albumin (≥4.2 vs <4.2 mg/dL), BMI (≥25 vs <25 kg/m2), and metabolic syndrome components. After adjusting for all possible confounders, interactions of the variables with adiponectin were evaluated through the addition of interaction terms. The trend for the mean adiponectin level among patients with varying numbers of metabolic syndrome components was also verified. A *P* value of <0.05 was considered significant. All statistical analyses were conducted using IBM SPSS Statistics for Windows version 22.0 (IBM SPSS Inc., Chicago, IL, USA).

## Results

### Participant characteristics

Table 1 summarizes the baseline characteristics of 196 patients stratified into two groups based on the presence of metabolic syndrome. This cohort had a mean age of 65.3 ±13.6 years and mean eGFR of 29.8 ± 12.8 mL/min/1.73 m^2^, and 66.3% of the study population was male. The median baseline adiponectin level was 29.4 (interquartile range, 13.3–108.7) μg/mL. Patients with metabolic syndrome had a significantly higher prevalence of gout and HTN as well as higher BMI, waist circumference, Hb, HDL, and TG levels. During the mean follow-up period of 5 years (66.5 ±17.7 months), 48 (24.5%) incident cases of ESRD were observed and 33 (16.8%) deaths occurred. In the analysis of the association of adiponectin with metabolic factors, the adiponectin concentration decreased progressively with increasing numbers of metabolic syndrome components (*P* for trend = 0.03).

**Table 1:** Clinical characteristics of patients according to metabolic syndrome.

Characteristics	All	Metabolic syndrome		*p*
	(n = 196)	No (n = 82)	Yes (n = 114)	
Adiponectin (μg/ml)	29.4 (13.3–108.7)	48.9 (16.3–113.1)	19.4 (11.4–55.4)	0.002
**Demographics**				
Age (year)	65.3 ± 13.6	67.3 ± 13.2	63.8 ± 13.8	0.078
Male gender (%)	130 (66.3%)	51 (62.2%)	79 (69.3%)	0.299
Smoking history (%)	39 (19.9%)	13 (15.9%)	26 (22.8%)	0.229
Hyperuricemia (%)	66 (33.7%)	21 (25.6%)	45 (39.5%)	0.043
Hypertension (%)	146 (74.5%)	52 (63.4%)	94 (82.5%)	0.003
Cardiovascular disease (%)	31 (15.8%)	10 (12.2%)	21 (18.4%)	0.239
Systolic blood pressure (mmHg)	136 ± 19	132 ± 19	139 ± 18	< 0.001
Diastolic blood pressure (mmHg)	80.4 ± 12.1	77.4 ± 12.8	78.9 ± 14.4	< 0.001
BMI (kg/m^2^)	24.1 ± 4.1	21.6 ± 3.0	25.9 ± 3.7	< 0.001
Waist circumference (cm)	85.8 ± 10.1	79.8 ± 8.0	90.2 ± 9.2	< 0.001
**Biochemical parameters**				
Albumin (g/dL)	4.2 ± 0.3	4.2 ± 0.3	4.3 ± 0.3	0.215
Hemoglobin (g/dL)	11.7 ± 2.0	11.2 ± 1.9	12.0 ± 2.1	0.014
Fasting glucose (mg/dL)	100.6 ± 11.0	95.8 ± 8.6	104.1 ± 11.3	< 0.001
HbA1c (%)	5.8 ± 0.4	5.6 ± 0.3	5.9 ± 0.4	< 0.001
Triglyceride (mg/dL)	117 (85–172)	94 (71–117)	154 (97–209)	< 0.001
Total cholesterol (mg/dL)	185 ± 38	184 ± 40	185 ± 37	0.947
HDL (mg/dl)	44.9 ± 14.1	51.8 ± 14.7	39.9 ± 11.3	< 0.001
LDL (mg/dl)	104.2 ± 28.7	103.3 ± 28.0	104.8 ± 29.3	0.718
CRP (mg/l)	2.0 (0.7–4.9)	1.0 (0.6–3.2)	2.4 (0.9–6.1)	0.015
eGFR (mL/min/1.73 m^2^)	29.8 ± 12.8	30.1 ± 12.6	29.6 ± 13.1	0.787
Phosphate (mg/dL)	3.8 ± 0.8	3.8 ± 0.7	3.9 ± 0.9	0.824
Uric acid (mg/dL)	7.4 ± 1.6	7.1 ± 1.7	7.5 ± 1.6	0.105
UPCR (mg/g)	530 (181–1371)	475 (194–1271)	506 (141–1363)	0.767
**Metabolic syndrome components**				
Metabolic factor	2.8 ± 1.3	1.5 ± 0.6	3.7 ± 0.7	< 0.001
Waist criteria	86 (43.9%)	8 (9.8%)	78 (68.4%)	< 0.001
BP criteria	172 (87.8%)	63 (76.8%)	109 (95.6%)	< 0.001
Sugar criteria	96 (49.0%)	16 (19.5%)	80 (70.2%)	< 0.001
HDL criteria	127 (64.8%)	29 (35.4%)	98 (86.0%)	< 0.001
TG criteria	68 (34.7%)	8 (9.8%)	60 (52.6%)	< 0.001
**Clinical outcomes**				
RRT	48 (24.5%)	21 (25.6%)	27 (23.7%)	0.720
RRT or 50% decline in eGFR	87 (44.4%)	38 (46.3%)	49 (43.0%)	0.709
All-cause mortality	33 (16.8%)	19 (23.2%)	14 (12.3%)	0.065

Data expressed as the mean ± standard deviation, median (interquartile range), or count (percentage).

Abbreviations: BMI, body mass index; HbA1c, glycated hemoglobin; HDL, high-density lipoprotein; LDL, low density lipoprotein; CRP, C-reactive protein; eGFR, estimated glomerular filtration rate; UPCR, urine protein-to-creatinine ratio; RRT, renal replacement therapy.

**Fig 1:**
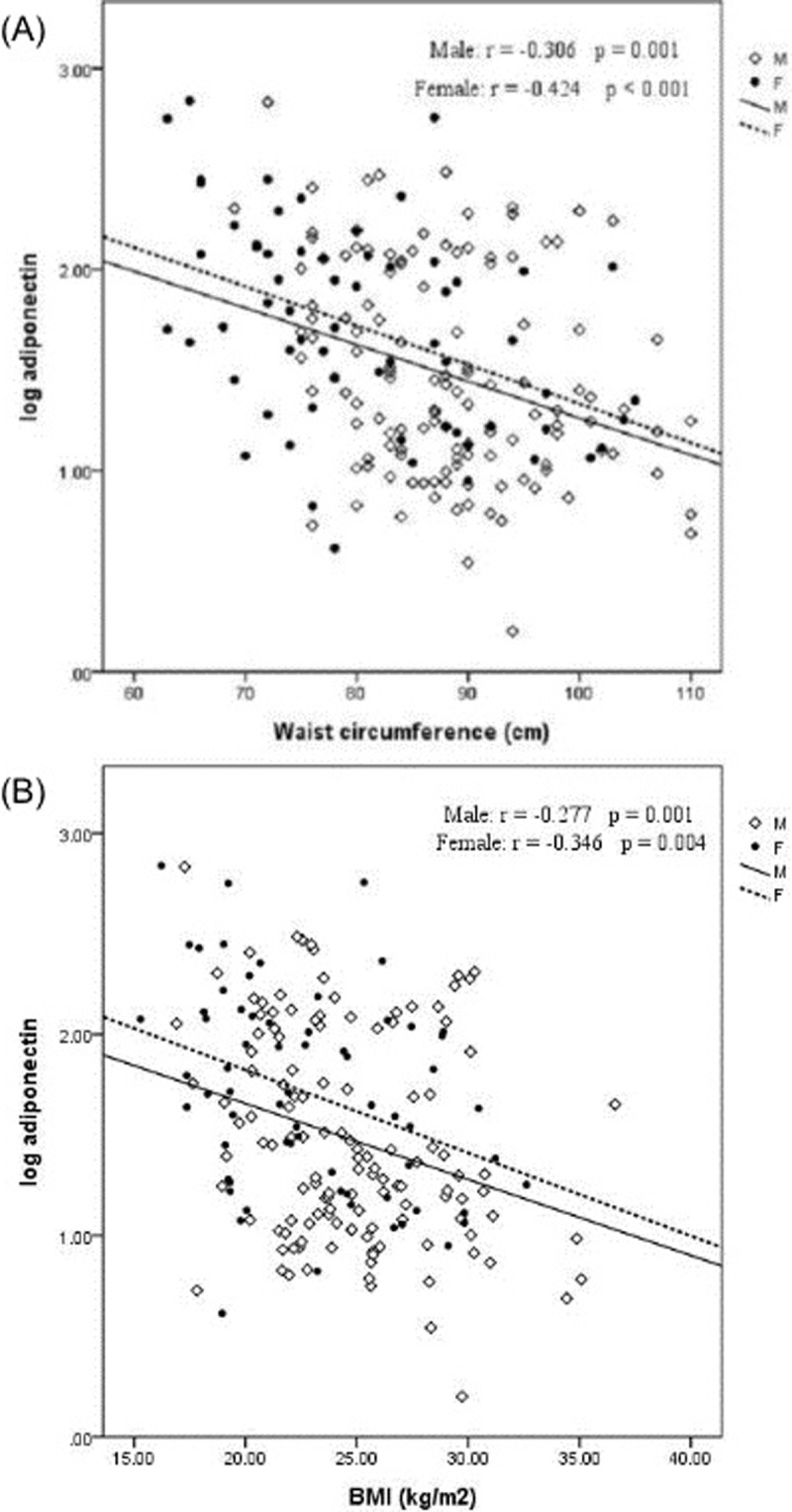
Correlations of adiponectin with adiposity index. (A) Correlations of adiponectin with BMI (B) Correlations of adiponectin with waist circumference.

Regarding sex differences in adiponectin level have been established in published studies [31–33], our cohort showed that adiponectin was higher (*P* = 0.03) in female patients (97.2±132.5 μg/mL) than in male patients (62.2±88.0 μg/mL). In addition, we separately evaluated correlation between adiponectin and adiposity covariates (BMI and waist circumference) according to sex. The unadjusted adiponectin level was strongly and inversely correlated with BMI (*r* = -0.29; *P* < 0.001) and waist circumference (*r* = -0.35; *P* < 0.001; Fig 1A and 1B) to a similar extent among both male and female patients.

### Relationship between clinical and biochemical variables and log-transformed adiponectin

Table 2 shows the result of multivariable linear regression of relevant clinical and biochemical covariates with log-transformed adiponectin as the dependent variable. However, collinearity between BMI and waist circumference was detected by Spearman rank correlation (r = 0.841; P < 0.001); formal testing showed a tolerance value (variance inflation factor) of 4.617. Therefore, we evaluated the linear regression in a separate model including different adiposity index (waist circumference or BMI). In the fully adjusted model, log-transformed adiponectin had a significantly negative relationship with waist circumference (β = −0.015; *P* < 0.001), BMI (β = −0.020; *P* < 0.04), MBP (β = 0.007; *P* = 0.01), HDL (β = 0.007; *P* = 0.03), and eGFR (β = −0.011; *P* = 0.002). The R2 value of the goodness-of-fit of this adjusted model was 0.27.

**Table 2:** Multivariable linear regression for determinants of adiponectin levels (log transformed).

	All		
Variables	β	95% CI	*p*-value
Age (years)	0.005	0.000 to 0.011	0.051
Male vs. female	0.096	-0.066 to 0.259	0.244
Waist circumference (cm)	-0.015	-0.022 to -0.007	<0.001
MBP	0.007	0.002 to 0.012	0.011
AC sugar	-0.002	-0.008 to 0.004	0.508
HDL	0.007	0.001 to 0.012	0.026
Log TG	-0.115	-0.459 to 0.228	0.503
Albumin	-0.096	-0.314 to 0.121	0.385
Hb	-0.037	-0.080 to 0.005	0.084
Log CRP	-0.006	-0.125 to 0.114	0.927
eGFR (10ml/min/1.73 m^2^)	-0.113	-0.185 to -0.041	0.002
Log UPCR	-0.120	-0.268 to 0.029	0.113
Adiposity index*			
Waist circumference	-0.015	-0.022 to -0.007	<0.001
Or BMI	-0.020	-0.039 to -0.001	0.040

* Linear regression in separate model including different adiposity index

Abbreviations are the same as in Table 1.

### Adiponectin concentration and its relationship with clinical outcomes

Multivariable Cox proportional hazard regression analysis was used to examine whether adiponectin is independently associated with clinical outcome risks (Table 3). In the unadjusted model, the HRs for each one-unit increase in the log-transformed adiponectin level (continuous variable) were 1.98 (95% CI, 1.17–3.39; *P* = 0.01) for RRT and 1.77 (95% CI, 1.19–2.63; *P* = 0.005) for composite renal outcomes. The estimates for log-transformed adiponectin became stronger with increment adjustment for demographic factors, BMI, and singular components of metabolic syndrome in models 1 and 2. With full adjustment (model 3) for laboratory parameters specific to ESRD (albumin, Hb, CRP, eGFR, and UCPR), the significant association of increasing adiponectin with renal outcome risks remained unchanged: HRs of 2.08 (95% CI, 1.08–4.02; *P* = 0.03) for RRT and 1.66 (95% CI, 1.03–2.65; *P* = 0.04) for composite renal outcome. Although the association between adiponectin and all-cause mortality reached significance in the unadjusted model (*P* = 0.04), the association disappeared after additional adjustment for variables.

**Table 3:** Baseline adiponectin level (continuous variable, log transformed) and outcomes.

	RRT		Composite renal outcome		All-cause mortality	
	HR (95%CI)*	*P*	HR (95%CI)*	*P*	HR (95%CI)*	*P*
Unadjusted	1.98 (1.17–3.39)	0.01	1.77 (1.19–2.63)	0.005	1.98 (1.05–3.72)	0.04
Adjusted						
Model 1^b^	1.94 (1.10–3.43)	0.02	1.69 (1.11–2.59)	0.02	1.10 (0.53–2.27)	0.80
Model 2^c^	2.08 (1.17–3.72)	0.01	1.78 (1.15–2.75)	0.01	1.04 (0.49–2.22)	0.91
Model 3^d^	2.08 (1.08–4.02)	0.03	1.66 (1.03–2.65)	0.04	0.93 (0.41–2.10)	0.86

*Per unit increase in log-transformed adiponectin

^b^ Adjusted for age, gender, BMI, gout

^c^ Adjusted for model 1 covariates plus metabolic syndrome criteria: (1) central obesity (waist circumference ≥ 90 cm in men or ≥ 80 cm in women, according to the ethnic criteria for Asians); (2)TG ≥ 150 mg/dL; (3) HDL cholesterol < 40 mg/dL in men and < 50 mg/dL in women; (4) fasting glucose ≥ 100 mg/dL or previously diagnosed with type 2 DM; (5) BP ≥ 130/85 mmHg or on treatment for hypertension

^d^ Adjusted for model 2 covariates plus Hb, Albumin, CRP, eGFR, UPCR

Values expressed as hazard ratios (HR) and 95% confidence interval (CI).

**Fig 2:**
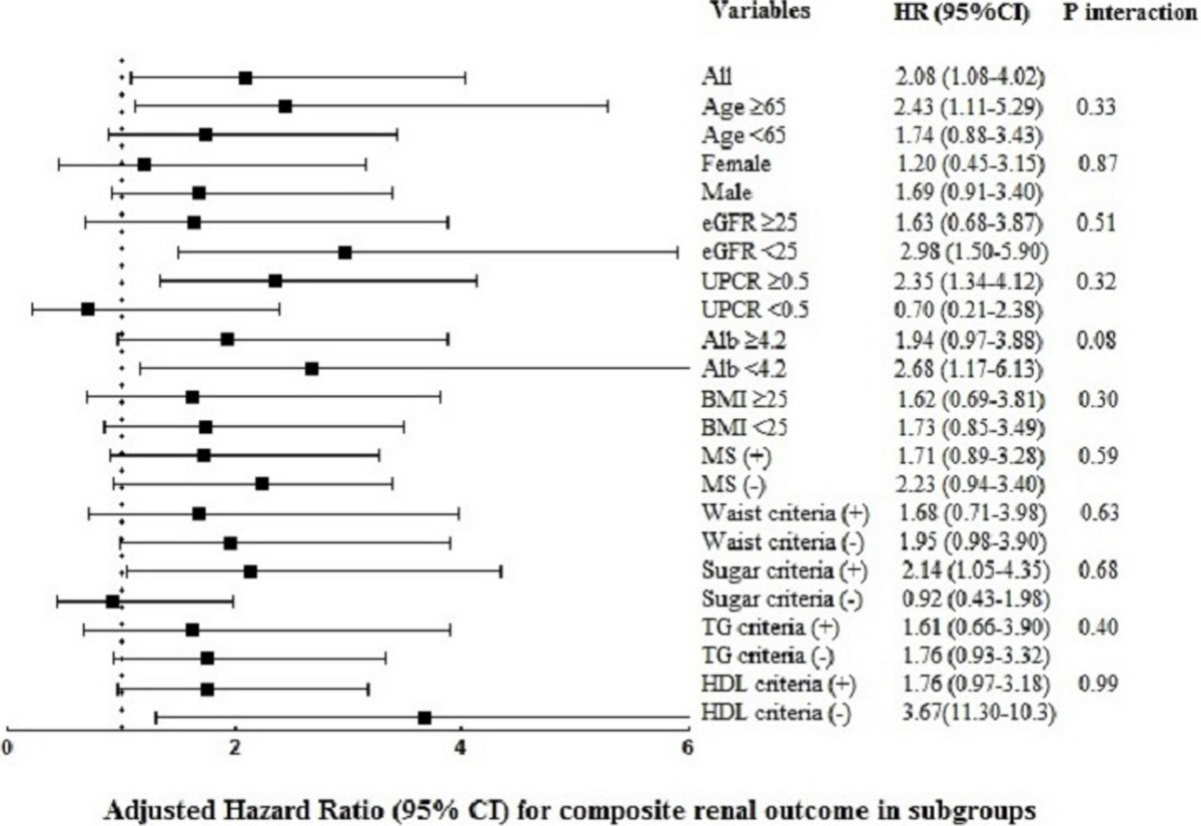
Subgroup analysis for the association between adiponectin level (log transformed) and the risk of composite renal outcome.

We did not explore significant effect modification of the association between adiponectin and composite renal outcome in different subgroups (Fig 2), including age, sex, eGFR, UPCR, albumin, BMI and presence of metabolic syndrome (*P* for interaction = 0.33, 0.87, 0.51, 0.32, 0.08, 0.30, and 0.59, respectively). Even for the interaction of singular criteria of metabolic syndrome, the result revealed nonsignificant.

## Discussion

In the present cohort of patients with non-diabetic CKD, the high adiponectin level was markedly associated with an increased risk of renal outcomes independent of BMI, metabolic syndrome, and conventional CKD risk factors. This finding remained consistent within subgroups of age, sex, metabolic syndrome, BMI, albumin, proteinuria, and eGFR status. In addition, higher adiponectin levels were correlated with lower BMI, waist circumference, and eGFR, which confirmed previously reported data in CKD patients[9, 10, 16, 18].

Adiponectin exists in the plasma as three major complexes: a low-molecular-weight trimer, a middle-molecular-weight hexamer, and a high-molecular-weight (HMW) isoform. Adiponectin activates AMP-activated protein kinase (AMPK) and peroxisome proliferator-activated receptor (PPAR)-α pathways which mediate its beneficial effect on insulin sensitivity via two major receptors (AdipoR1 and AdipoR2) [34]. Regarding its anti-inflammatory role, adiponectin suppresses macrophage function[35], inhibits the nuclear factor κB (NF-κB) pathway[36], interferes endothelial adhesion molecules[37], and induces the expression of anti-inflammatory cytokines[38]. Adiponectin levels are mainly determined by the abdominal visceral fat mass, with an inverse proportion found between them[39]. Obesity decreases both circulating adiponectin levels and adiponectin receptor expression, resulting in the reduction of downstream signaling[40]. Similar to the correlation found in the populations without CKD, our study indicated significant negative correlations between two adiposity surrogates (BMI and waist circumference) in CKD patients, which is in agreement with previous reports. Both BMI and waist circumference can predict abdominal adiposity in CKD patients [41]. Yoon et al. investigated obesity-linked metabolic dysregulation and showed that hypoadiponectinemia is independently associated with the presence of metabolic syndrome in CKD patients, which is definite evidence of the relationship of adiponectin with kidney function[10]. In the present study, we found that the adiponectin level tended to decrease as the number of metabolic syndrome components increased.

Elevated circulating adiponectin has been found in various stages of CKD and in maintenance dialysis. Adiponectin is negatively related to eGFR across mild to advanced CKD[16, 18]. Our study showed fairly consistent findings in multivariate analysis. Based on available knowledge, there are several potential explanations for the negative correlation. First, urinary adiponectin clearance diminishes accompanied with kidney dysfunction, as urinary adiponectin levels are inversely correlated with GFR[42]. The HMW adiponectin isoform, rather than the LMW isoform, has been reported to be a more biologically active component and is accumulated in ESRD[43, 44]. Second, the expression of adiponectin mRNA and AdipoR1 is upregulated in visceral and subcutaneous fat tissue in ESRD patients despite concomitant elevated circulating adiponectin levels[45]. Moreover, postreceptor block in adiponectin receptor signaling has been found[46]. All these findings imply that when kidney function deteriorates, the paradoxical increase in adiponectin may be attributed to the resistance effect of adiponectin rather than only being a reflection of decreased renal elimination. Third, adiponectin rise may represent a compensatory response to counteract chronic inflammation and atherosclerosis, which both characterize CKD status. In addition, the existence of insulin resistance even in early CKD stages may result in an adaptive response to the altered metabolic profiles in terms of elevated adiponectin[15, 47]. Fourth, enhanced production of adiponection in CKD may also reflect a physiologic response for mitigating kidney injury. In human renal tubular cells, adiponectin has been found to attenuate the adverse effects of angiotensin II by inhibiting NADPH oxidase, NF-κB activity, and fibronectin expression[48]. However, clinical studies have indicated the relationship between adiponectin and albuminuria has a biphasic pattern[49], with a positive association detected in more advanced albuminuria[50]. Therefore, elevated adiponectin might be a beneficial compensatory mechanism to protect against renal injury.

The clinical implications of adiponectin are more complex in CKD. Contrary to the favorable anti-inflammatory, insulin-sensitizing, and cardioprotective effects of adiponectin in healthy subjects, varying results have been obtained for the association between adiponectin and adverse outcomes in CKD. Several studies have indicated an reverse association that a high, rather than a low, adiponectin level predicts adverse CV outcomes and all-cause mortality in CKD[6, 21, 22], and this association is attenuated in ESRD. Zoccali et al. did not find any relationship between high adiponectin and increased mortality in HD patients[31]. Similarly, Dreschler et al. found no association of adiponectin with all-cause mortality in the largest HD cohort to date[25]. In addition, although adiposity might be an important modifier of adverse outcomes in CKD[7, 23], Rhee et al. supported that higher adiponectin remained associated with mortality in HD patients even under comprehensive adjustment for body anthropometry[6]. Therefore, in addition to adiposity, the consequences of persistent inflammation and malnutrition might be implicated in the mechanism through which high adiponectin promotes poor outcomes and eGFR decline. In our study, adiponectin failed to predict overall mortality. The result suggested that multiple biological pathways of adiponectin and differences in statistical adjustment or population characteristics, including age, sex, comorbidity, inflammation and nutritional status, or disease severity might involve in the result.

There is limited and conflicting evidence for the association of adiponectin with kidney outcomes. Using the MDRD database, Menon et al. did not reveal a direct correlation between adiponectin and the risk of kidney failure among 820 patients with GFR ranging between 13 and 55 ml/min/1.73 m^2^ (mean GFR 33 ± 12 mL/min/1.73 m^2^)[21]. Conversely, Kollerits et al. identified that high adiponectin predicted CKD progression in men but not in women among 177 non-diabetic patients with mild to moderate CKD (mean GFR 64 ± 39 mL/min/1.73 m^2^)[19]. Even in type 1 diabetes, adiponectin was an independent risk factor for progression from macroalbuminuria to ESRD, irrespective of HbA1c or eGFR status[51]. Although the eGFR of our patients (29.8 ± 12.8 mL/min/1.73 m^2^) was similar to that of patients in the MDRD study, our findings are in contrast with theirs. The potential reasons for the discrepancy might be that our patients were older and relatively non-obese (median BMI 23.6 kg/m^2^), which perhaps highlighted aggravating PEW and inflammatory status in our patients. On the other hand, we also found that the association between adiponectin and ESRD risk was not modified by BMI or metabolic syndrome components. This finding particularly suggests that weight loss or adiposity cannot fully account for poor outcomes. BMI reflects not only fat mass but also muscle mass; the negative relationship of BMI with adiponectin raises the possibility of PEW in higher adiponectin, as previously reported[9]. Indeed, both chronic inflammation and PEW are important determinants of CKD outcomes. Moreover, we did not find sex differences for CKD progression as the study reported by Kollerits et al., which BMI was used for metabolic syndrome diagnosis instead of waist circumference and women with metabolic syndrome was less frequent though[19]. Our study is the first to establish the association of adiponectin with renal outcomes in a comprehensive analysis including metabolic syndrome components, and the results were consistent in different subgroups.

Our study has several limitations that should be acknowledged. First, we did not measure serial adiponectin levels during follow-up, and the isoforms of adiponectin were not differentiated, as the HWM isoform has been reported to be the most biologically active form. Second, apart from BMI and albumin, nutritional indices, such as muscle mass and dietary intake indicators, were not fully included to evaluate PEW status. Therefore, inadequate adjustment of malnutrition markers for the outcome may contribute to residual confounding. Third, the sample size of 65 female patients was relatively small (33.5%) compared with the sample size of male patients. Imbalance in the groups might be likely to interpret the loss of sex-specific associations in the present study.

In conclusion, our study revealed higher adiponectin was associated with a higher risk of ESRD, independent of various ESRD-related variables, including BMI and metabolic syndrome components. In addition, adiponectin was closely correlated with BMI and metabolic syndrome components. Future larger studies are warranted to elucidate the exact predictive effects of adiponectin, particularly different isoforms, among the CKD population in analyses including relevant risk factors.
